# Improving antibody affinity through *in vitro* mutagenesis in complementarity determining regions

**DOI:** 10.7555/JBR.36.20220003

**Published:** 2022-03-28

**Authors:** Wei Ye, Xiaoyu Liu, Ruiting He, Liming Gou, Ming Lu, Gang Yang, Jiaqi Wen, Xufei Wang, Fang Liu, Sujuan Ma, Weifeng Qian, Shaochang Jia, Tong Ding, Luan Sun, Wei Gao

**Affiliations:** 1 Jiangsu Key Lab of Cancer Biomarkers, Prevention and Treatment, Collaborative Innovation Center for Personalized Cancer Medicine, Key Laboratory of Human Functional Genomics of Jiangsu Province, National Health Commission Key Laboratory of Antibody Techniques, School of Basic Medical Sciences, Nanjing Medical University, Nanjing, Jiangsu 211166, China; 2 The Affiliated Suzhou Hospital of Nanjing Medical University, Suzhou Municipal Hospital, Gusu School, Nanjing Medical University, Suzhou, Jiangsu 215001, China; 3 Department of Biotherapy, Nanjing Jinling Hospital, Nanjing, Jiangsu 210002, China

**Keywords:** antibody engineering, phage display, affinity maturation

## Abstract

High-affinity antibodies are widely used in diagnostics and for the treatment of human diseases. However, most antibodies are isolated from semi-synthetic libraries by phage display and do not possess *in vivo* affinity maturation, which is triggered by antigen immunization. It is therefore necessary to engineer the affinity of these antibodies by way of *in vitro* assaying. In this study, we optimized the affinity of two human monoclonal antibodies which were isolated by phage display in a previous related study. For the 42A1 antibody, which targets the liver cancer antigen glypican-3, the variant T57H in the second complementarity-determining region of the heavy chain (CDR-H2) exhibited a 2.6-fold improvement in affinity, as well as enhanced cell-binding activity. For the I4A3 antibody to severe acute respiratory syndrome coronavirus 2, beneficial single mutations in CDR-H2 and CDR-H3 were randomly combined to select the best synergistic mutations. Among these, the mutation S53P-S98T improved binding affinity (about 3.7 fold) and the neutralizing activity (about 12 fold) compared to the parent antibody. Taken together, single mutations of key residues in antibody CDRs were enough to increase binding affinity with improved antibody functions. The mutagenic combination of key residues in different CDRs creates additive enhancements. Therefore, this study provides a safe and effective *in vitro* strategy for optimizing antibody affinity.

## Introduction

An antibody is a powerful immune molecule with a clear mechanism of action. At present, antibodies are widely used in biological research as well in diagnostics and for frontline therapeutics^[1–3]^. Phage display is a widely used and is a powerful technology that allows the display of antibody fragments on the surface of filamentous bacteriophages infecting *E. coli*^[4]^*.* This approach uses an *in vitro* selection process that does not have to rely on immunization, and can make use of entirely human gene repertoires^[5–7]^.

Through our previous research, we developed a series of therapeutic and neutralizing antibodies which included the 42A1 antibody against glypican-3 (GPC3) and the I4A3 antibody targeting receptor-binding domains (RBDs) in the severe acute respiratory syndrome coronavirus 2 (SARS-CoV-2) spike glycoprotein. GPC3 is a cell surface oncofetal protein that is considered an immunotherapeutic target for hepatocellular carcinoma^[8–10]^. The SARS-CoV-2 spike RBD is responsible for the binding of the virus to the ACE2 in host cells, and thus this domain is considered as the main target for neutralizing antibodies^[11–13]^. Both 42A1 and I4A3 were isolated from the Tomlinson I and J libraries as has been described in our open-access patents^[14–15]^. 42A1 specifically recognizes the surface membrane tumor antigen GPC3 and has the potency for further translational development, including therapeutic antibodies, immunotoxins and chimeric antigen receptor T cells. As a neutralizing antibody to SARS-CoV-2, I4A3 performs an effective viral blocking activity and therefore prevents viral invasion. These two antibodies are excellent prospects for a number of diseases and provide additional value for optimization.

The quality of the antibody library is essential for *in vitro* antibody screenin*g*. Four types of libraries can now be identified through sources of antibody repertoires, *i.e.*, naïve, immune, synthetic, and semi-synthetic^[16]^. In semi-synthetic libraries, there is a combination of naturally derived and synthetically designed parts, and the ratio of these parts varies under different scenarios^[17–18]^. The Tomlinson I and J libraries are widely-used semi-synthetic libraries, which constitute a stable IGHV3-23 framework and the kappa IGKV1-39 framework with randomized positions in complementarity-determining region 2 (CDR2) and CDR3^[19]^. The size of the Tomlinson I library is 1.47×10^8^ different scFv fragments, while that of the Tomlinson J library is 1.37×10^8[19]^. Generally speaking, antibody affinities from phage libraries are proportionally determined according to the size of the library: up to 10 nM for libraries with 10^7^ to 10^8^ clones, and up to 0.1 nM for the best libraries with over 10^10^ members^[20]^. Due to the diversity of CDR designs in semi-synthetic libraries, which is just one or two CDRs diversity, the screened antibodies exhibit only moderate affinity^[16,20]^. Therefore, both 42A1 and I4A3 appear to have sensitive antigen-binding specificity and potency function, although their antigen-binding affinity requires improvement to satisfy translational requirements. Therefore, engineering the affinity of these antibodies through *in vitro* assaying is necessary.

Current antibody affinity maturation methods usually include two mutagenesis strategies: stochastic and targeted mutagenesis^[16]^. In stochastic mutagenesis, the sequences for the variable fragment (Fv) can be mutated randomly through error-prone polymerase chain reaction (PCR) or by introducing mutator bacterial strains^[21]^. By contrast, targeted mutagenesis introduces diversity in predictable positions, mainly ones which contribute to antigen binding, and are also workable by window mutagenesis or site-directed mutagenesis^[22–23]^. As the specificity and binding affinity of antibodies are predominantly determined through CDRs, it would seem logical that engineering CDRs will directly contribute to improving antibody properties^[22,24]^.

Computational approaches have been widely accepted as tools for antibody engineering. These methods have also been implemented to assist researchers screening libraries and also to optimize pharmacokinetic properties such as affinity, specificity and stability^[25–27]^. In the current study, we performed a series of point mutations within a single CDR or combined different CDRs to improve affinity. After *in vitro* affinity maturation, 42A1 and I4A3 obtained improved affinity and were simultaneously accompanied by elevated cell binding or neutralizing activity. Therefore, our work provided a safe and effective *in vitro* strategy to optimize antibody affinity. Furthermore, the optimized whole human antibody can be further used for clinical development.

## Materials and methods

### Cell culture

A431, HEK293T, and 293T cells were purchased from American Type Culture Collection (USA). Cells were maintained at 37 °C, 5% CO_2_, in DMEM (HyClone, USA) supplemented with penicillin (100 U/mL), streptomycin (100 μg/mL) (HyClone), and 10% fetal bovine serum (FBS; VACCA, USA). A431 cells were engineered to ensure high expression of GPC3 by transfection with a plasmid encoding full-length GPC3.

CHO-K1 cells were maintained in DMEM/F-12 (HyClone) supplemented with 10% FBS (VACCA), 100 U/mL penicillin, and 0.1 mg/mL streptomycin (HyClone) and were incubated in 5% CO_2_ at 37 °C. CHO-K1 cells were engineered to highly express ACE2 by transfection with a plasmid encoding full-length ACE2 protein.

### Preparation of the 42A1 mutation plasmids

The 42A1 antibody sequence was cloned into the pIT2 vector. 42A1 mutation plasmids were created using inverse PCR (iPCR) to introduce site-directed mutagenesis in CDR-H2^[28]^. We designed partially overlapping primers containing 190 mutations at 10 sites of CDR-H2. All PCR products were gel-purified, and digested with the restriction enzyme *Dpn* I (TaKaRa, Japan) to remove the methylated template plasmid and for transformation into TG1 competent cells by standard heat shock protocol (Weidi Biotechnology, China). The collected clones were sequenced to confirm the correctness of mutations.

### Preparation of the I4A3 site-saturated random plasmid libraries

The sequence of the I4A3 antibody was cloned into the pIT2 vector. Partially overlapping primers with the NNK randomization (N randomizing with all four nucleotides and K introducing only G or T) were designed to introduce random mutations in 15 sites of the two CDRs, *i.e.*, CDR-H2 and partial CDR-H3. Then, each site was constructed as a site-saturated random plasmid library. Each library was gel-purified, digested with the restriction enzyme *Dpn* I to remove the methylated template plasmid and transformed into TG1 electrocompetent cells by standard electrocompetent transformation protocol.

### Generation of full-length antibodies

For protein purification, the heavy chain variable region and light chain variable region sequences of parental antibodies (and their mutations) were amplified by adding IL-2 signal peptide and were inserted into expression vectors, pFUSE-CHIg-HG1 and pFUSE2-CLIg-hk (Invivogen, USA), respectively. 293T cells were transiently transfected with plasmids carrying the antibody heavy chain and the light chains at a 1:1 ratio.

After transfection, the supernatant was harvested daily for five consecutive days. Supernatant collected was then pooled and clarified by centrifugation (3000 *g* for 5 minutes, 4 °C) followed by filtration through a 0.45 μm filter. Affinity chromatography was used to purify expressed monoclonal antibodies using a Protein A affinity column (GE Healthcare, USA) able to bind to the Fc fragment. Purified antibodies were buffer-exchanged into phosphate buffer saline (PBS), concentrated using Amicon Ultra-4 10 kDa centrifugal filter units (Millipore Sigma, USA) and stored at 4 °C until use.

### Purification of GPC3-hFc and SARS-CoV-2-RBD hFc

In order to generate GPC3-hFc and SARS-CoV-2 RBD-hFc proteins, the sequence of GPC3 (Q25-S550) or RBD (P330-V524) was fused to a human Fc tag, cloned into a pFUSE vector and transfected into 293T cells followed by media collection and purification using a Protein A affinity column. Purified proteins were buffer-exchanged into PBS, concentrated using Amicon Ultra-4 10 kDa centrifugal filter units and stored at 4 °C until use.

### Periplasmic extraction of single-chain variable fragment variants from bacteria

TG1 periplasmic extracts containing 42A1 or I4A3 scFv variants were produced using the vector pIT2. Clones were then added to 900 μL of freshly prepared 2×YT medium supplemented with 100 μg/mL ampicillin in a 96-deep well plate and incubated overnight at 37 °C with shaking cultivation. The next day, these plates were centrifuged at 3000 *g* for 10 minutes at 4 °C, then sedimented cells were resuspended with 900 μL of freshly prepared 2×YT medium containing 100 μg/mL ampicillin and 1 mmol/L isopropyl β-D-1-thiogalactopyranoside, and incubated for 4 hours at 30 °C with gentle shaking cultivation. Completely removed culture media resuspend the cell pellet in 300 μL of TES buffer (0.2 mol/L Tris-HCl, 0.5 mol/L sucrose, 1 mmol/L EDTA, 1 mg/mL lysozyme) by vortex mixing. These were then incubated on ice for 30 minutes before being centrifuged at 3000 *g* for 20 minutes. The supernatant with a volume of 200 μL was then transferred to new plates.

### Enzyme-linked immunosorbent assaying

Enzyme-linked immunosorbent assaying (ELISA) was used to analyze antibody affinity curves: GPC3-hFc protein or SARS-CoV-2-RBD-hFc protein (5 μg/mL) was used to coat ELISA wells at 4 °C overnight. Bovine serum albumin (BSA, 5 μg/mL) was used as control. Wells were blocked with PBS containing 3% milk for 0.5 hours at 37 °C. The antibodies were added to the wells and incubated at 37 °C for 0.5 hours. After washing with PBST 3 times, goat anti-human kappa chain HRP antibody (Life Tech, USA) was added to the wells and incubated at 37 °C for 0.5 hours. Tetramethyl benzidine (TMB) and H_2_SO_4_ were added to detect the OD value at 450 nm.

To capture ELISA for screening high-affinity mutants, goat anti-c-myc Ab (Bethyl-A190-204A, 1 μg/mL) in the PBS were coated in the ELISA wells with 100 μL/well and incubated overnight at 4 °C. After blocking with 3% PBST milk, all interval washes were performed three times with PBST. Plates were washed and scFv periplasma, from the periplasm of TG1, was added to ELISA wells with 100 μL/well and incubated at room temperature for 1 hour. After washing, GPC3-hFc or SARS-CoV-2-RBD-hFc protein (2 μg/mL) was added to the wells with 100 μL/well and incubated at room temperature for 1 hour. Plates were washed three times, then goat anti-human HRP antibody (Jackson IR, 109-036-098) was added before being incubated at room temperature for 1 hour. TMB and H_2_SO_4_ were added to detect the OD value at 450 nm.

### Flow cytometry

A single-cell suspension of A431-GPC3 was incubated with 5 µg/mL of the indicated antibodies for 1 hour on ice before being incubated with a 1:200 dilution of anti-human PE antibody, for 42A1 and its mutant (Thermo, USA) for 1 hour on ice. Cells were analyzed using BD FACSCalibur (BD Biosciences, USA).

### Pseudovirus neutralization assay

To generate SARS-CoV-2 pseudovirus, we engineered an infectious molecular clone of vesicular stomatitis virus by replacing the endogenous glycoprotein with a SARS-CoV-2 spike in the lentiviral packaging system^[29–30]^, which were then co-transfected with HEK293T cells and the pLVX-EGFP-Luciferase reporter gene. At 48 hours after transfection, the pseudovirus supernatant was harvested, clarified, filtered, and tittered as 10^5^ pfu/mL.

Neutralization assays were performed by incubating the pseudovirus with serially diluted antibodies at 37 °C for 1 hour. All antibodies and viruses were diluted with 10% FBS-DMEM. Then, the pseudoviral-antibody mixture was added to seeded CHO-ACE2 cells in 96-well plates and incubated at 37 °C for 48 hours. The half-maximal inhibitory concentration (IC_50_) of each antibody was determined by measuring luciferase activity.

### Statistical analysis

 Representative results were obtained through at least three independent experiments. All group data (except those indicated) are expressed as means with corresponding standard deviations (SD). All statistical analyses were conducted using GraphPad Prism 8.0. Further analysis of means was conducted using two-tailed Student's *t*-test. A *P*-value of <0.05 was established as the threshold for statistical significance.

## Results

### Isolation of human monoclonal antibodies 42A1 and I4A3 by phage display

**Figure 1:**
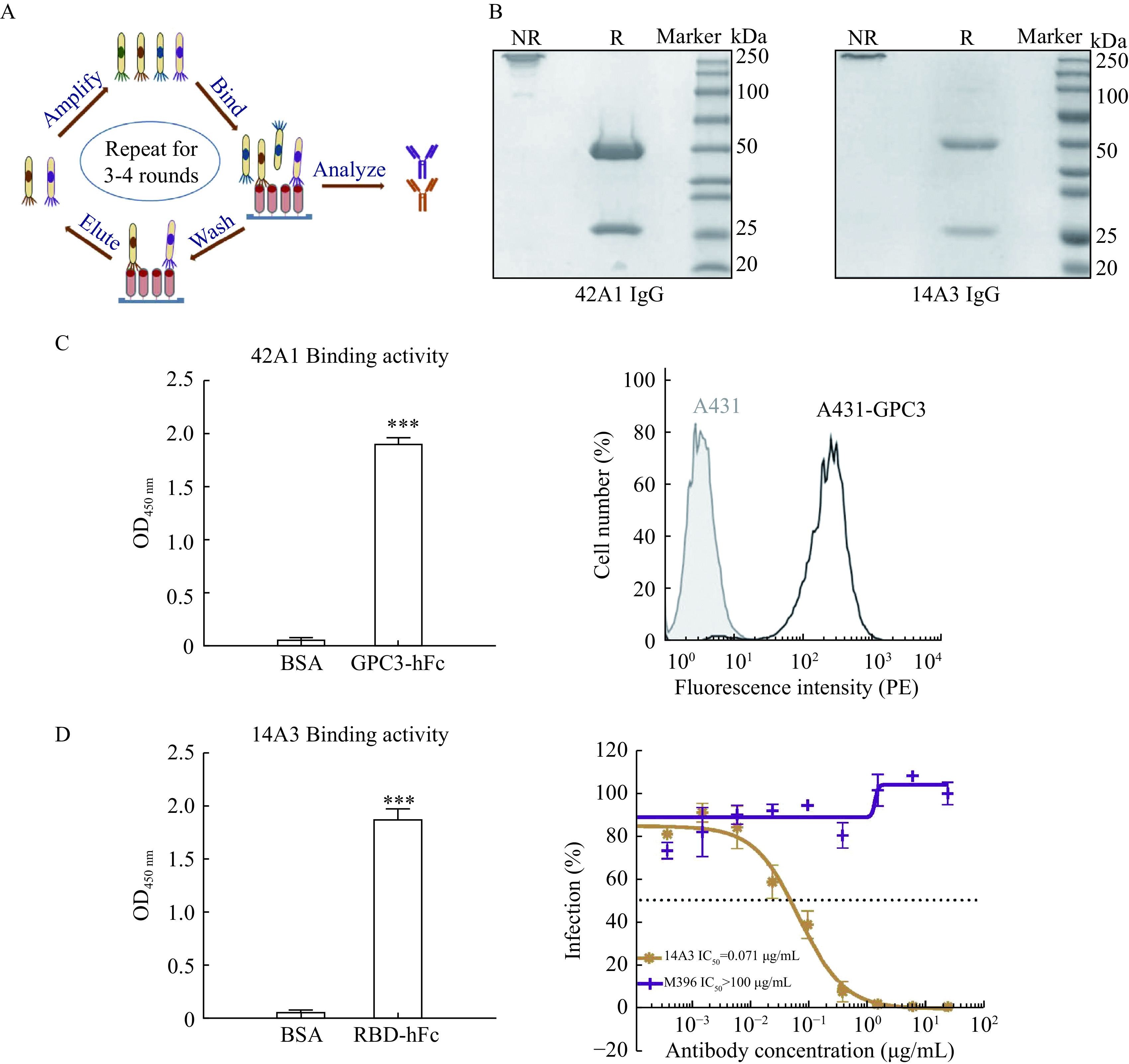
Screening of human monoclonal antibody targeting GPC3 and SARS-CoV-2 RBD by phage display.

To generate human monoclonal antibodies against the target antigen, we performed phage display screening on the Tomlinson I and J libraries^[14–15,19]^. After several rounds of panning to enrich the specific binder, antibody 42A1 against GPC3 and antibody I4A3 against SARS-CoV-2 RBD were identified (***Fig. 1A***). We then constructed vectors in order to purify antibodies in the IgG format for further analysis (***Fig. 1B***).

4A21 IgG showed specific binding activity to purified GPC3 protein and GPC3-positive cells (***Fig. 1C***). Similarly, the I4A3 antibody recognized purified SARS-CoV-2 RBD and could neutralize SARS-CoV-2 pseudovirus efficiently (***Fig. 1D***). Since these two antibodies both exhibited good specificity and functionalities, we decided to perform *in vitro* affinity maturation to further improve their binding activity.

### Improving antibody affinity by point mutations within CDR

It has been reported that affinity-enhancing mutations tend to cluster around positions where *in vivo* somatic mutations often occur^[31–32]^. Somatic hypermutation does not occur randomly within immunoglobulin V genes but preferentially targeted certain nucleotide positions, which are most frequently located in CDRs, especially in CDR2 or CDR3^[33–35]^. Due to design limitations of Tomlinson I and J libraries^[19]^, both 42A1 and I4A3 contained short CDR-H3 and relatively long CDR-H2. Therefore, we first constructed a series of point mutation variants in CDRs of 42A1 and I4A3 to improve their affinity.

**Figure 2:**
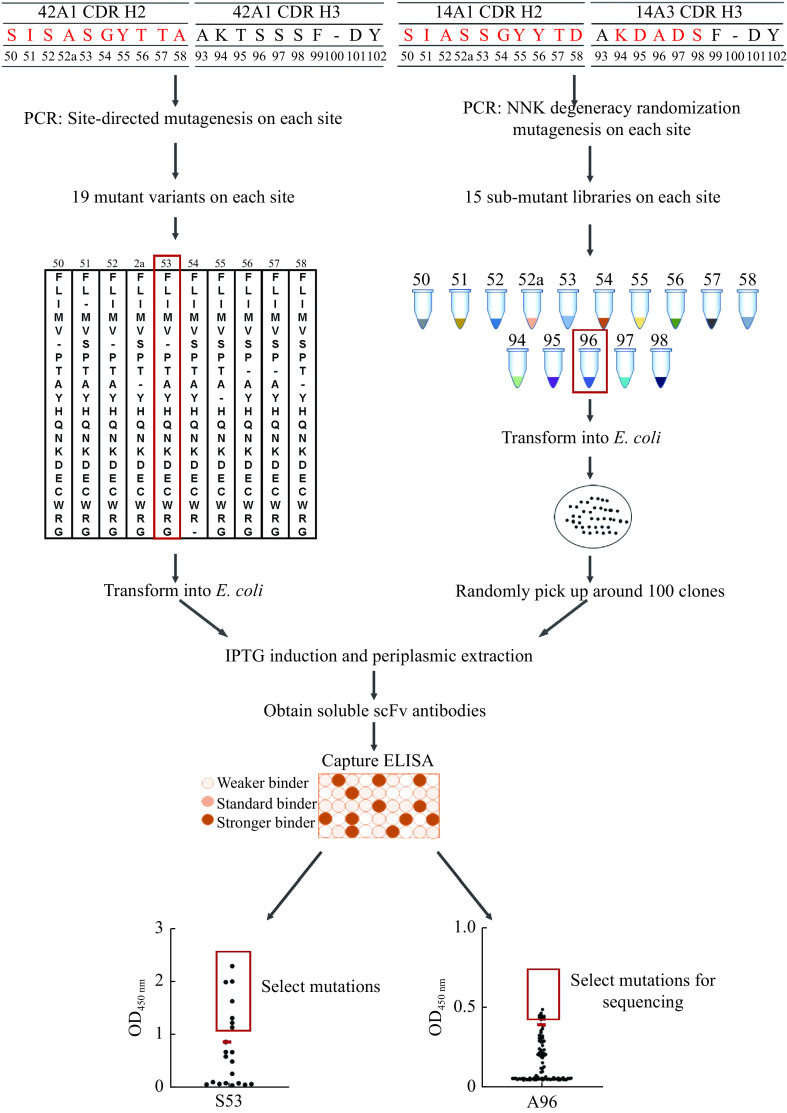
Overview strategy of affinity maturation for 42A1 and I4A3 by single point mutations in CDR.

We performed two different strategies to randomize CDRs of antibodies to achieve better affinity through mutation assaying (***Fig. 2***). For 42A1, we performed site-directed mutation PCR to mutate the single site of CDR-H2 to the remaining 19 amino acids. All 19 variants were sequenced, expressed in a soluble-scFv format, and their antigen-binding activity was compared with wild-type antibody through ELISA capturing. Those which exhibited improved antigen-binding activity were selected for further evaluation. All the 10 sites in 42A1 CDR-H2 were optimized according to this strategy, and we constructed 190 variants (19 mutants for each site) in total. This method appeared to work well but lacked efficiency since we had to construct all 190 mutants and sequenced them even though most did not influence the improvement of antigen-binding.

For the I4A3 antibody, we implemented another strategy that involved introducing random mutation by NNK degeneracy PCR (N randomizing with all four nucleotides and K introducing only G or T). For each site in CDR-H2 and partial site of CDR-H3, we constructed a sub-mutant library by NNK degeneracy PCR and transformed the sub-mutant library into *E. coli*. Then, we randomly picked up around 100 clones, expressed them in soluble-scFv format, and compared their antigen-binding activity with wild-type antibodies by ELISA capture. Clones with improved antigen-binding activities were selected and sequenced. We constructed 15 sub-mutant libraries corresponding to the 15 sites of CDR-H2 and CDR-H3 of I4A3 in total and successfully obtained the antibody variants.

**Figure 3:**
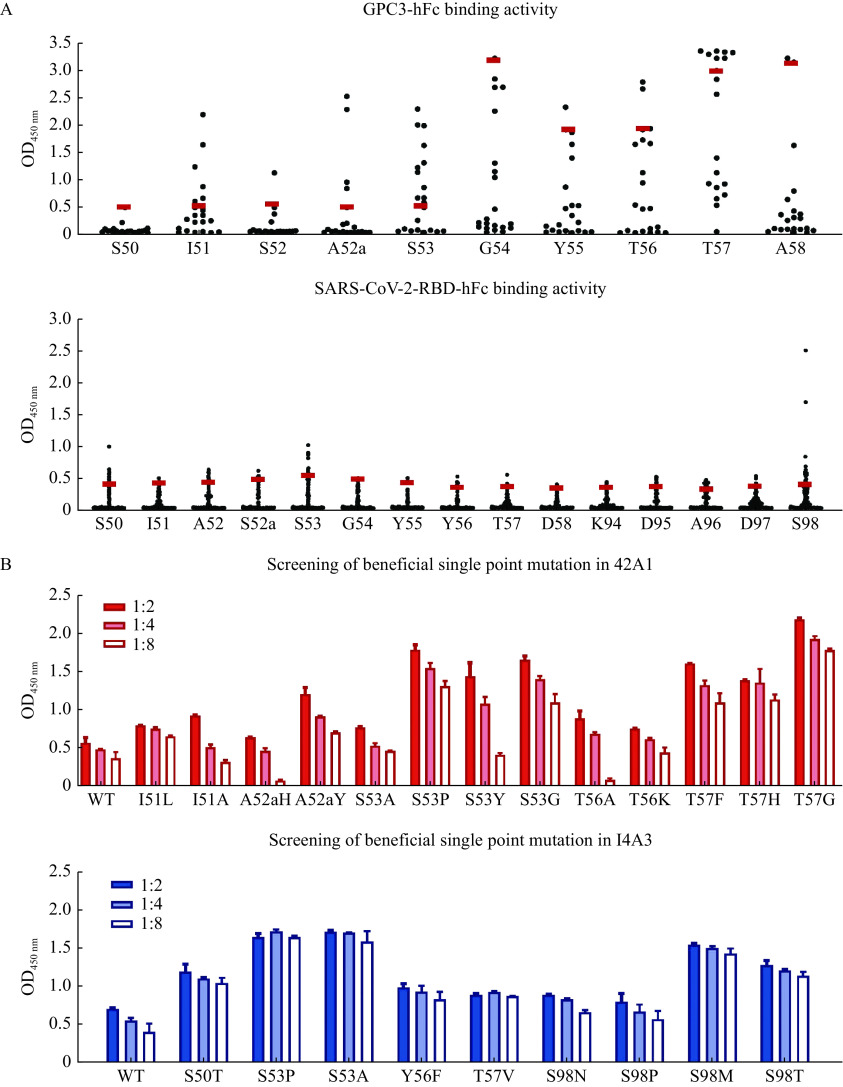
*In vitro* affinity maturation of the 42A1 and I4A3 by single point mutations in CDR.

After the first round of screening, we found that some point mutations on site I51, A52a, S53, T56, and T57 of 42A1 exhibited robust binding to purified GPC3 protein. Part of the point mutations on sites S50, S53, Y56, T57, and S98 of I4A3 showed elevated binding to purified SARS-CoV-2 RBD proteins (***Fig. 3A***). Among the mutant variants raised from these sites, those with increased antigen-binding activity were selected. After gradient dilution, the antigen-binding activity of these variants was further investigated. Seven mutants of 42A1, *i.e.*, A52aY, S53P, S53Y, S53G, T57F, T57H, and T57G as well as five mutants of I4A3, *i.e.*, S50T, S53P, S53A, S98M, and S98T were identified (***Fig. 3B***).

### Single point mutation in CDR caused improved affinity and functions of antibody

**Figure 4:**
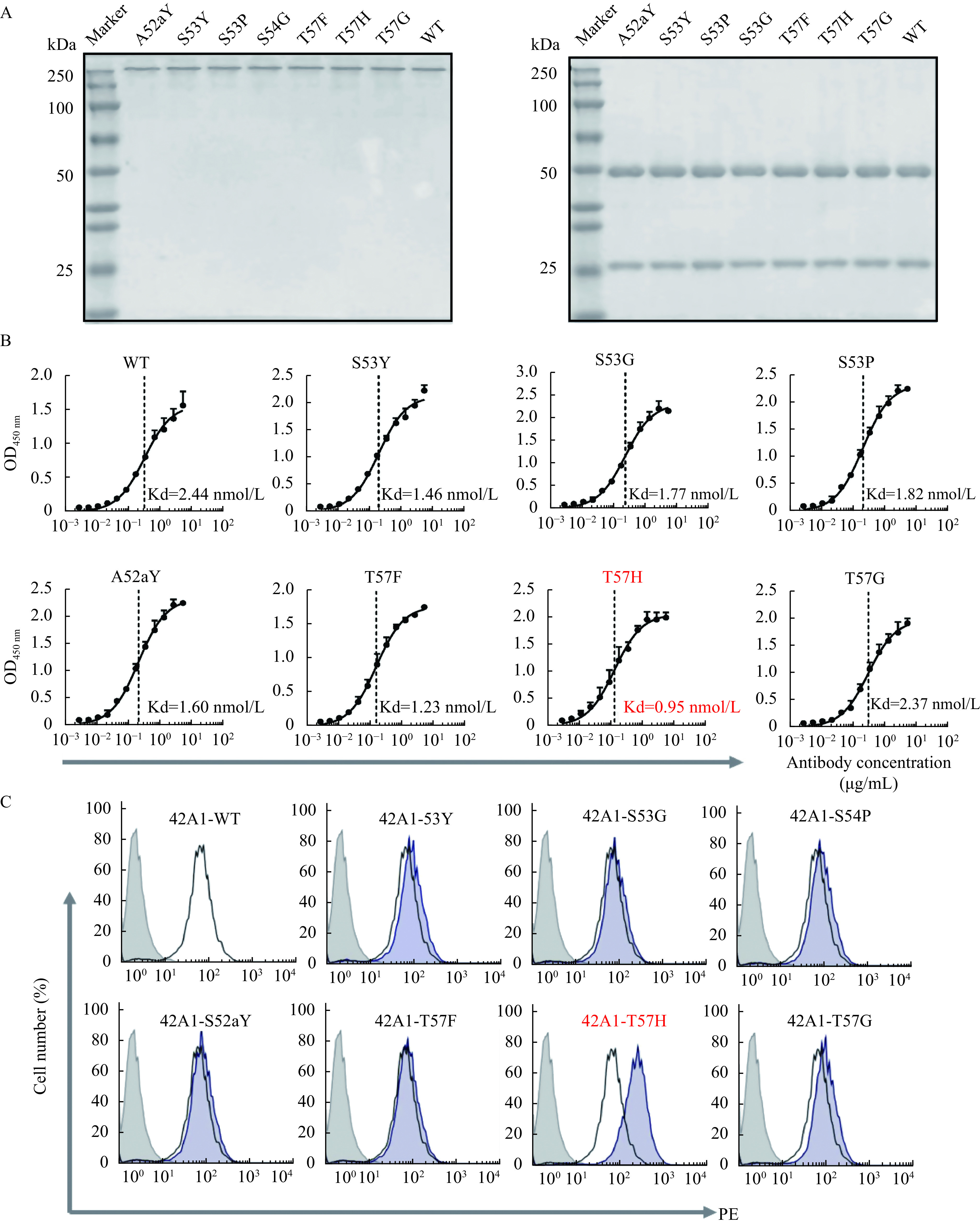
Antigen-binding activity and GPC3-positive cell recognition evaluation of affinity-matured 42A1 antibodies.

To further characterize mutant antibodies, we converted all beneficial mutants of antibody 42A1 into an IgG format, expressed in 293T cells and then they were purified (***Fig. 4A***). We then assessed their antigen-binding activity and functionality. The seven mutant variants of 42A1 showed varying degrees of improved potency. Among which the binding affinity of 42A1-T57H was almost 2.6-fold more than that of parental 42A1 on purified GPC3 (***Fig. 4B***). All mutant antibodies still maintained their binding capacity on GPC3-positive cells, but only 42A1-T57H exhibited improved cell-binding activity. This indicates that this might work as a valid variant with potency to develop antibody drugs (***Fig. 4C***).

**Figure 5:**
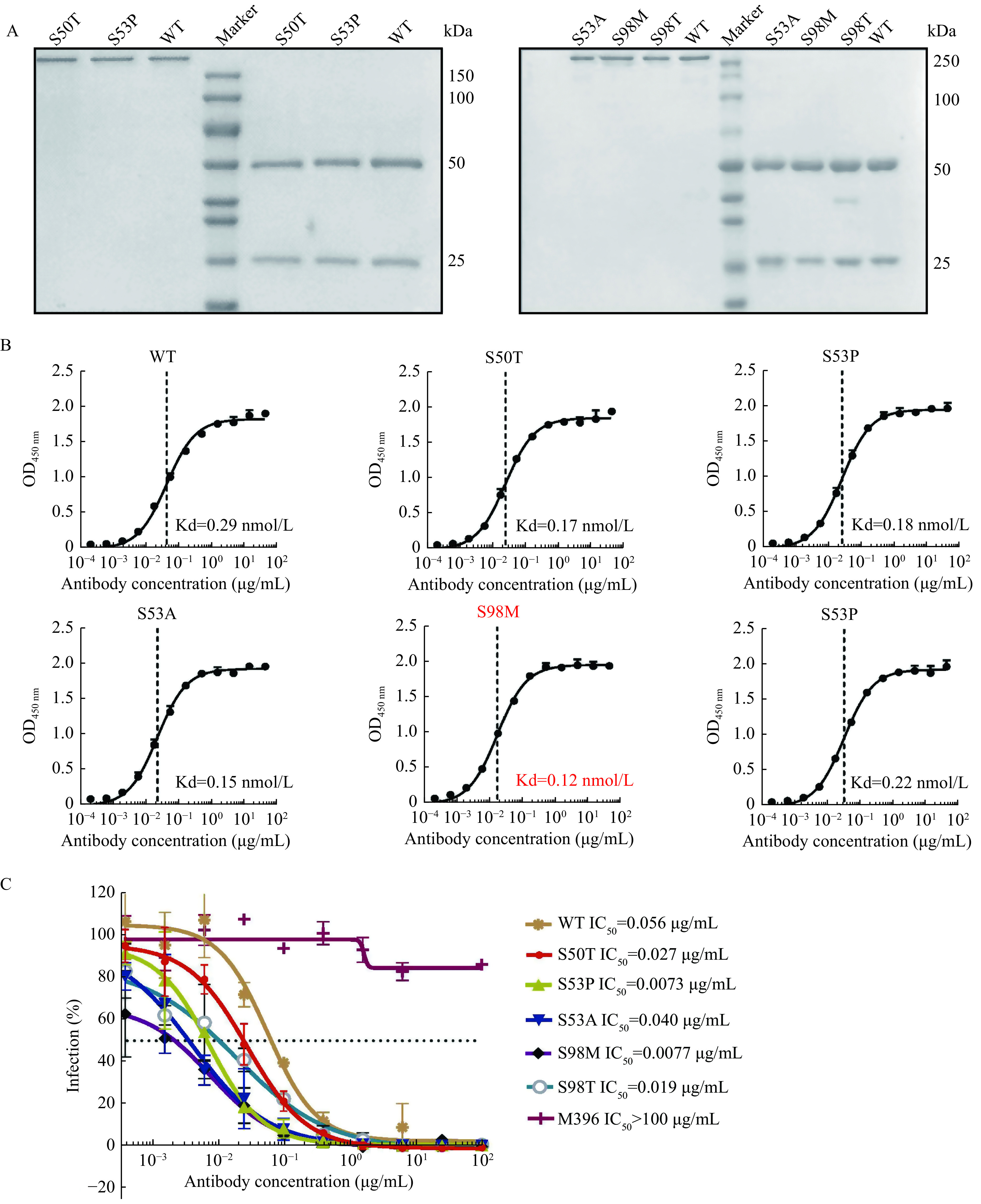
Antigen-binding activity and pseudovirus neutralizing effect evaluation of affinity-matured I4A3 antibodies.

On the other hand, after purifying all selected single point mutants of antibody I4A3 as IgG (***Fig. 5A***), we compared the "bind-ability" of each antibody against the SARS-CoV-2 RBD protein. The affinity of I4A3-S98M exhibited approximately a 2.4-fold improvement (***Fig. 5B***). We then investigated the neutralization activity of these variants against SARS-CoV-2 infection using a pseudovirus assay. As could be expected, most mutants of I4A3 showed improved neutralizing capability, and the affinity-matured antibody I4A3-S98M demonstrated the most dramatic improvement (>7-fold) compared with its parental antibody (***Fig. 5C***). Altogether, we successfully improved the affinity and function of our antibody by introducing single point mutations in CDR.

### Combining multiple point mutations in I4A3 CDR-H2 and CDR-H3 further improved affinity

**Figure 6:**
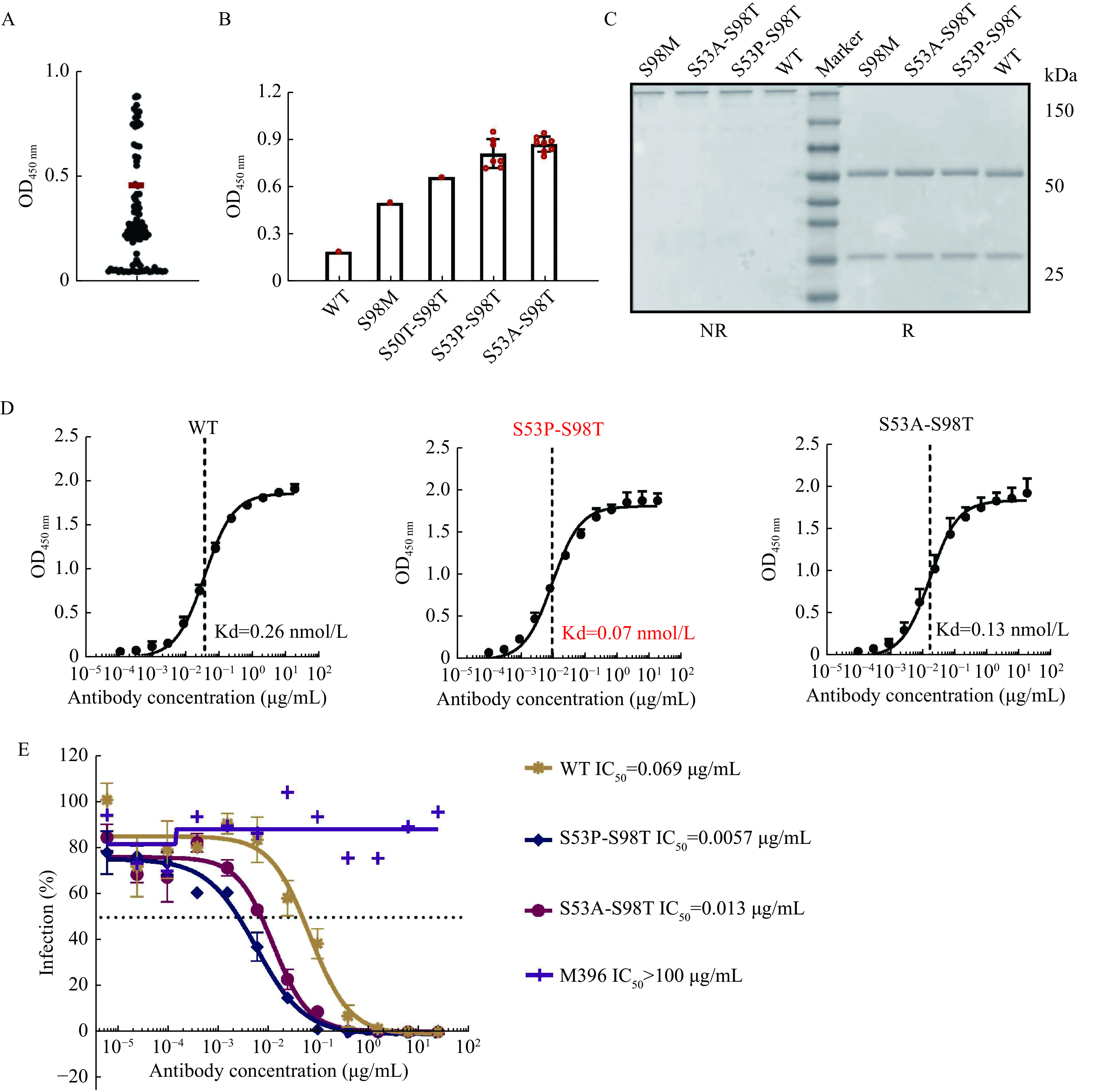
Combination of beneficial single point mutations to gain a further improved neutralizing activity of I4A3.

It has been reported that individual CDRs of the light and heavy chain are sequentially modified using site-specific mutagenesis and the best candidates are subsequently combined^[36–37]^. Although not always predictable, the additive effects could result in affinity increases ranging from two to three orders of magnitude^[38]^. After the first round of optimization, we then considered combining selected point mutations in CDR-H2 and CDR-H3 of I4A3 to achieve further improvement. As was expected, the combination of these five single point mutations resulted in an additional boost (***Fig. 6A***). In the obtained beneficial clones, S53A-S98T and S53P-S98T showed the most significant improvement when assessed using periplasmic extraction of scFv variants from bacteria (***Fig. 6B***).

We then expressed these combined mutations and purified them as IgG (***Fig. 6C***). The affinity of combined mutation increased more than a single point mutation, and the variant I4A3 S53P-S98T exhibited approximately a 3.7-fold improvement compared to the parental I4A3 (***Fig. 6D***). Similarly, the variant I4A3 S53P-S98T with the best affinity among all joint-mutant variants displayed the most potent neutralizing activity which achieved an approximate 12-fold improvement (***Fig. 6E***). The S53P-S98T combination, which utilizes optimized interactions of the single matured S53P and S98T, resulted in additional improvement in neutralization.

## Discussion

Phage display is a technology established for generating human monoclonal antibodies. This approach is an *in vitro* technology that confers the potential for generating antibodies from phage libraries against any conceivable molecule of sufficient size while overcoming limitations of *in vivo* immunization^[5,7]^. With full-length purified GPC3 and the RBD domain of SARS-CoV-2 as antigens, we screened the Tomlinson I and J libraries and obtained specific antibody fragments 42A1 and I4A3, from each respectively. In this study, we successfully optimized affinities by *in vitro* assaying under different scenarios.

Semi-synthetic libraries usually have combinations of natural and synthetic diversity. That is, they are often created to increase natural diversity while maintaining a certain level of functional diversity^[20]^. Although the semi-synthetic libraries, *i.e.*, Tomlinson I and J libraries are sources of antibodies against a large number of different antigens, those antibodies normally exhibit only moderate affinity due to limited diversity^[19–20]^. Optimizing the CDR sequence in order to improve affinity has been validated by engineering antibodies against various antigens^[22,34,36–38]^. Constructing a library of mutant variants in phage format provided a fast and convenient way to identify which potent binders bypass the protein purification process.

The choice of an affinity maturation strategy is related to both the magnitude of affinity increases and the probability of epitope conservation. Therefore, we first optimized antibody affinity by introducing a single site mutation in CDR. We felt this approach might have less chance of influencing epitope. Our results showed that T57H mutation in CDR-H2 of 42A1 and S98M mutation in CDR-H3 of I4A3 both brought 2- to 3-fold improvement in affinity. This suggests that the strategy implemented is feasible; however, we were unable to obtain the optimized variants from the original phage library. This might be due to the limited diversity in the Tomlinson I and J libraries. Additionally, the initial phage library may or may not contain antibody variants with point mutations for those selected. Moreover, the enrichment of specific antibodies in phage display screening relies, not only, on its binding property but also depends on the expression level of each specific antibody. Therefore, the other possibility is that these mutant antibody variants might not have the expression priority compared to the original one selected.

The first-round screening of single site mutants enabled us to identify residues that were most likely to become potential candidates for combined multiple mutations. After the second round of optimization by combining two mutant sites of CDR, we successfully achieved a 12-fold improvement in neutralizing activity of I4A3 compared to a 7-fold increase in activity gained from single point mutations. This is an impressive enhancement based on two-point mutations in CDR. It is possible that antibody affinity may be significantly improved by applying multiple rounds of mutation. However, this strategy still only provides us with a limited range of affinity improvement, perhaps because our work on sequence optimization was without changing the residue number in CDRs. Therefore, we postulate the need to identify more opportunities for enhancements by optimizing the length of CDRs.

It has been reported that some therapeutic antibodies (Tremelimumab, Ipilimumab, and Afasevikumab) with similar sequence identity to SARS-CoV-2 neutralizing antibodies (C002, HbnC3tlpl_G4, and COV2-2015) have the potential for cross-reactivity. This potential exhibits a high binding overlap with the same epitope regions on SARS-CoV-2 spike proteins^[39]^. However, despite 42A1 and I4A3 having relatively high, similar sequences, they did not display cross-reactivity, suggesting that the observed limited CDR difference guarantees recognition specificity. Alternatively, recently published computational results have provided insights into antibody affinity optimization by analyzing the structural characteristics of antigen-antibody interactions^[40]^. This suggests that analyzing the sequence and structural characteristics of a series of optimized antibodies selected in the current study by computational calculations might also be useful for obtaining antibodies with improved affinity.

In summary, we performed a series of site-directed random mutagenesis within a single CDR or in combined CDRs to improve antibody affinity. After *in vitro* affinity maturation, 42A1 and I4A3 had improved affinity and were accompanied by elevated cell binding and neutralizing activity. Therefore, this study provides a safe and effective *in vitro* strategy which optimizes antibody affinity. These findings could be useful for therapeutics development although further research in to CDR length optimization is required.
